# Study of the anticorrosive behavior of samarium as a corrosion inhibitor in multilayer systems for aluminum alloy

**DOI:** 10.1038/s41598-023-30193-y

**Published:** 2023-02-23

**Authors:** José Antonio Cabello Mendez, Ailed Arguelles Rojas, José de Jesús Pérez Bueno, Yunny Meas Vong

**Affiliations:** 1 Centro de Investigación y Desarrollo Tecnológico en Electroquímica, S. C., Parque Tecnológico Querétaro-Sanfandila, Pedro Escobedo, C.P. 76703 Querétaro, Mexico; 2 Universidad Tecnológica del Centro de Veracruz, Av. Universidad 350, 94910 Cuitláhuac, Veracruz Mexico

**Keywords:** Corrosion, Materials science, Electrochemistry, Materials chemistry

## Abstract

This study shows a multilayer system based on samarium compounds as a corrosion inhibitor and a continuous SiO_2_ layer by atmospheric pressure plasma jet (APPJ) as a protective barrier for aluminim alloy AA3003. One of the main advantages of this new coating is that it does not require vacuum chambers, which makes it easy to incorporate into production lines for automotive and aeronautical components, etc. The deposit of samarium corrosion inhibitor was carried out by two methods for comparison, the immersion method and a novel method to deposit corrosion inhibitor by APPJ. The multilayer system generated was homogeneous, continuous, adherent, and dense. The electrochemical behavior shows that the samarium compound was completely oxidized on coatings by the immersion method and favors corrosion. The APPJ deposition method shows a protective behavior against corrosion by both samarium compounds and silica depositions. XPS analyses show that the amount of Sm(OH)^3^ increases by the APPJ method compared with the immersion method since the spectrum of O1s is mainly controlled by OH. It was determined that the best processing times for the electrochemical study of the multilayer system were 40 min for the immersion method and 30 s for the APPJ method for the layer of corrosion inhibitor. In the case of the SiO_2_ barrier layer by APPJ, the best time was 60 s of exposure to the plasma jet and this coating could reduce the corrosion of AA3003 by 31.42%.

## Introduction

Corrosion in metals is a major global problem that has affected the economics of the metallurgy industry. It is estimated that around 10 to 20% of world metal production is lost annually due to corrosion^1^ and this represents an annual expenditure of about 4% of the global gross domestic product^2^. However, not all the problems involved in corrosion are based on the materials, there are also problems of environmental pollution^3^. For example, the built environment materials are mainly affected by HNO_3_, SO_2_, and PM_10_ compounds influencing corrosion^4^. In addition, corrosion represents a severe problem in various areas such as pipes^5^, medicinal materials^6^, semiconductor packages^7^, automotive industry^8^, and construction^9^, among others.

Over time, different methods and techniques have been developed to control corrosion in metals, such as chrome plating^10^ and anodizing^11,12^ stand out as solutions for the control of this problem. Currently, the most studied methods are chromium conversion layers (CCL)^13^ or chromic acid anodizing (CAA)^12^, and tartaric sulfuric acid anodizing (TSA)^14^. However, these solutions are potentially toxic to the environment and even to human health since, in their processes, they use Cr(VI), which is highly noxious and has carcinogenic properties^15,16^. Consequently, the European Community has enacted restrictions on the use of heavy metals, including Cr(VI)^17,18^. Therefore, over the last few years to the present day, new technologies and methods have been sought that are more friendly to the environment and human health. Of these new technologies, in the field of corrosion, there is a trend toward developing environmentally friendly and low-cost corrosion inhibitors^19^. A corrosion inhibitor can be defined as a substance that, in low concentration, slow down the rate of corrosion^20^.

Despite its passivation layer, aluminum is susceptible to corroding in the presence of aqueous solutions containing ions, such as Cl^−^, Br^−^, or I^−^^21,22^. The corrosion problem in aluminum alloys can be addressed using corrosion inhibitors^23–27^. Hinton, Arnott, and Ryan stand out as the pioneers in introducing rare earth corrosion inhibition coatings as a sustainable option by depositing rare earth oxides on the surface of metals, such as Sm, Ce, La, Ne, and Pr, generating a protective film against corrosion^28–30^. Of the rare earth elements, cerium and samarium are the most commonly studied as corrosion inhibitors due to their abundance and efficiency^31,32^. Samarium is a rare earth element capable of forming samarium compound thin films to protect metals against corrosion^32,33^. Samarium has been used in conversion coating^34,35^ and alloys to improve corrosion prevention in aluminum^36,37^, magnesium^38,39^, and steel^40,41^. The principle of protection against corrosion using samarium as an inhibitor is based on the formation of oxides and hydroxides that are insoluble and block the cathodic sites, which slows down the corrosion rate^42^.

The protective effect of coatings on aluminum cast alloys has been extensively investigated in the literature^43–47^. The Atmospheric Pressure Plasma Jet (APPJ) is a surface treatment focused on preventing and protecting against corrosion. Likewise, it can reduce hydrophobicity, modifying the composition of the substrate surface and favoring the growth of passive oxide films. In addition, the coatings have better adhesion. Due to this set of characteristics, the APPJ technique has the potential to be used in the placement of multilayers that delay the phenomenon of corrosion^48^. Furthermore, this technique allows for cleaning and chemically modify the surface to increase the wettability making the substrate more hydrophilic, depositing fine and coarse particles in a reproducible way, activating surfaces, and changing the degree of hydrophobicity by varying the parameters of the plasma^48^.

This work compares two methods to deposit samarium, as a corrosion inhibitor, in a multilayer system using SiO_2_ as a barrier to protect the aluminum alloy AA3003 from corrosion. The corrosion inhibitor was deposited by two methods, the first was a method already reported in the literature, which consists of immersing the substrate in a bath with H_2_O_2_. The second method was a novel samarium coating obtained using the Atmospheric Pressure Plasma Jet.

## Materials and methods

**Figure 1:**
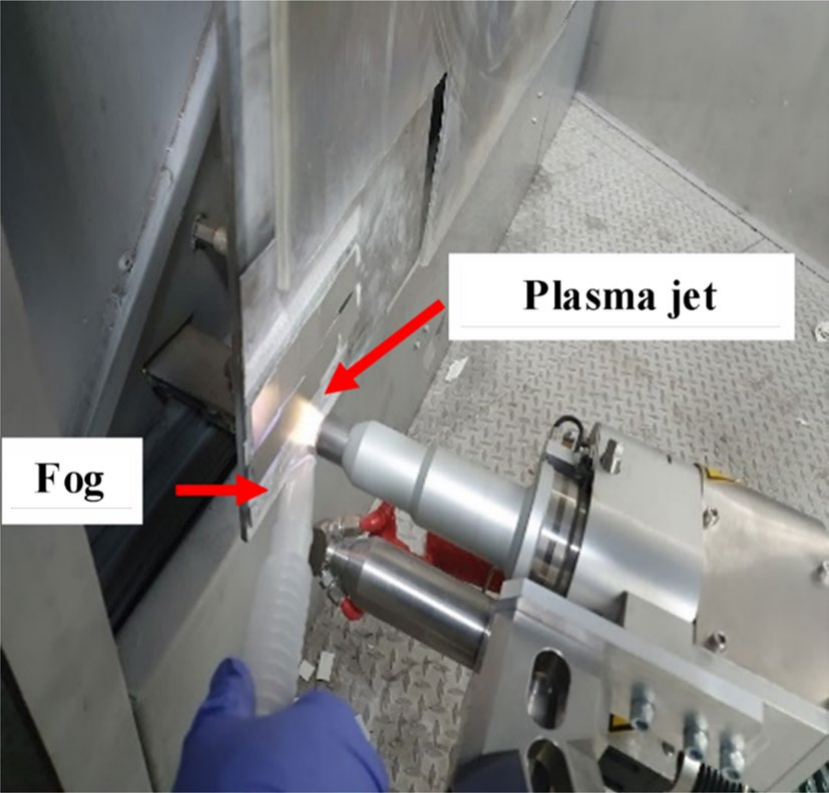
Experimental assembly of the technique for surface treatment and deposit of coatings by APPJ.

The substrate was aluminum 3003 washed with deionized water and acetone to remove impurities. The substrate composition was (Al 96.8–99 wt%, Cu 0.05–0.2 wt%, Fe 0.7 wt% max, Mn 1–1.5 wt%, Si 0.6 wt% max, Zn 0.1 wt% max, residuals each 0.05 wt% max, total 0.15 wt% max)^49^. All reagents used are reactive grade. The AA3003 aluminum plates were cleaned with deionized water and acetone (C_3_H_6_O, 99%, Karal). An Atmospheric Pressure Plasma Jet (APPJ) was used. The equipment has two heads “Plasma Cleaning” (*Openair-Plasma*®) and “Plasma coating” (*Plasma Plus*^®^)^50^. In this work, only the Plasma Cleaning head was used as a surface pre-treatment and for the generation of coatings through ultrasonic fog solutions. This fog was obtained with a Yue-Hua brand ultrasonic nebulizer, model WH-2000. Figure 1 shows the experimental assembly of the use of the APPJ system to get coatings, where ultrasonic fog (precursor) is incorporated into the plasma zone with the aid of a nozzle and using a distance of 2 cm between substrate and plasma jet. The plasma sprays the fog onto the substrate to form the coating.

Coatings of samarium were carried out with samarium sulfate 8.4 mM as a precursor (Sm_2_(SO_4_)_3_, 99.99%, Aldrich). The coatings of samarium species were made using two methods, immersion and atmospheric pressure plasma jet. For the immersion method, the dissolution was heated to 65 °C and 10% v/v hydrogen peroxide (30%) was added. The immersion times were 10, 20, 40, and 60 min. Subsequently, the treated samples were washed with deionized water. In the case of the APPJ method, the samples were treated with 0.5 and 1 min of exposition to the jet of plasma, using a distance of 2 cm between the sample and the plasma jet with the projection of a precursor fog. After depositing the samarium species layer, the barrier coating was applied.

For the second layer (barrier coating), samples were made with a layer using the *Openair-Plasma*® jet^50^. The distance between the plasma nozzle and the substrate surface was 2 cm. The exposition times were 50, 60, 70, 160, and 300 s with the projection of an ultrasonic fog of tetraethyl orthosilicate (TEOS) reagent grade (Sigma-Aldrich). The purpose of this layer was to serve as a physical barrier to delay corrosion. This type of coating has the characteristics of being continuous, homogeneous, dense, and adherent.

Before each treatment with APPJ, a pre-treatment of surface conditioning with *Openair-Plasma*^®^ was applied to the aluminum AA3003 for 30 s, where the substrates were placed at about 1.5 cm from the *Openair-Plasma*^®^ jet^50^. This pretreatment allows sample cleanup and increases adherence to the subsequent layer.

The samples were characterized by scanning electron microscopy (SEM), energy dispersive X-ray spectroscopy (EDS) microanalysis, and elemental mapping with a JEOL model JSM-6510 scanning electron microscope with built-in EDS. Micrographs were taken at 500×, 1000×, and 5000× using scale bars of 50, 10 µm, and 5 µm, respectively. A Keyence VHX-5000 series Digital Microscope was used. In this case, micrographs were taken at 500× and 2000× with a scale bar of 100 µm. The X-ray photoelectron spectroscopy (XPS) was conducted with a Thermo Scientific KAlpha^™^ + spectrometer, which used an Al Kα monochromatized X-ray source (hν = 1486.6 eV). The operating pressure was about 10^−9^ mBar, with a spot size of about 400 μm, energy step size of 20.0 eV, with a total of 10 scans, and erosion of about 15 s to reduce adsorbed atmospheric compounds. All acquired spectra were processed using a Gaussian-type peak based on NIST Standard Reference Database 20 4.1 and bibliographical references.

Aluminum AA3003 samples with a first samarium compound layer and the second SiO_2_ layer were electrochemically tested. In all techniques, the electrolyte was NaCl 3.5% and the solutions were bubbled with nitrogen to remove the oxygen in the electrolyte. The reference electrode was AgǁAgCl, and the counter electrode was graphite. Previously to each electrochemical technique, an open circuit potential measurement was conducted for 1 h to stabilize the thermodynamic system. For the electrochemical noise technique, the ZRA was measured for 15 min applying an external potential between two identical samples. A second-degree polynomial adjustment was used to remove the effect of the direct current component. The potential (σ_E_) and current (σ_I_) standard deviations were calculated. The noise resistance (Rn) was calculated with Eq. (1)^51^.

1 $${R}_{n}=\frac{{\sigma }_{E}}{{\sigma }_{I}},$$

where, Rn is the noise resistance in Ω, σ_E_ is the potential standard deviation in V, and σ_I_ is the current standard deviation in A. The pitting index (PI) was calculated with Eq. (2)^52^, where PI is the pitting index and RMS (I) is the mean quadratic root of the current in A.

2 $$\mathrm{PI}= \frac{{\upsigma }_{\mathrm{I}}}{\mathrm{RMS}(\mathrm{I})}.$$

The electrochemical impedance spectroscopy (EIS) technique was used to evaluate the frequency of the substrates and determine their resistance to corrosion. EIS measurements were performed at open circuit potential (OCP), with an amplitude of 10 mV and a frequency of 10^5^ to 1 Hz.

Cyclic potentiodynamic polarization (CPP) measurements were studied at 10 mV/s from initial potential (E_i_ =  − 0.3 V *vs*. OCP) to return potential (E_r_ = 0.6 V *vs*. OCP), and the final potential was 0 V *vs.* E_i_. The corrosion rate (CR), corrosion current (I_corr_), and corrosion potential (E_corr_) were determined with Tafel slopes, extrapolating the anodic and cathodic slopes. CR was calculated with Eq. (3)^53,54^, where CR is the corrosion rate in mm/year, K is a constant with a value of 3272 mm/cm per year, I_corr_ is the corrosion current (A), EW is the equivalent weight (g/eq), $\rho$ is the density (g/cm^3^), and A the sample area (cm^2^).

3 $$\mathrm{CR}=\mathrm{K }\frac{{\mathrm{i}}_{\mathrm{corr }}\mathrm{EW}}{\mathrm{\rho A}}.$$

## Results and discussion

### Samarium compound layers by immersion

**Figure 2:**
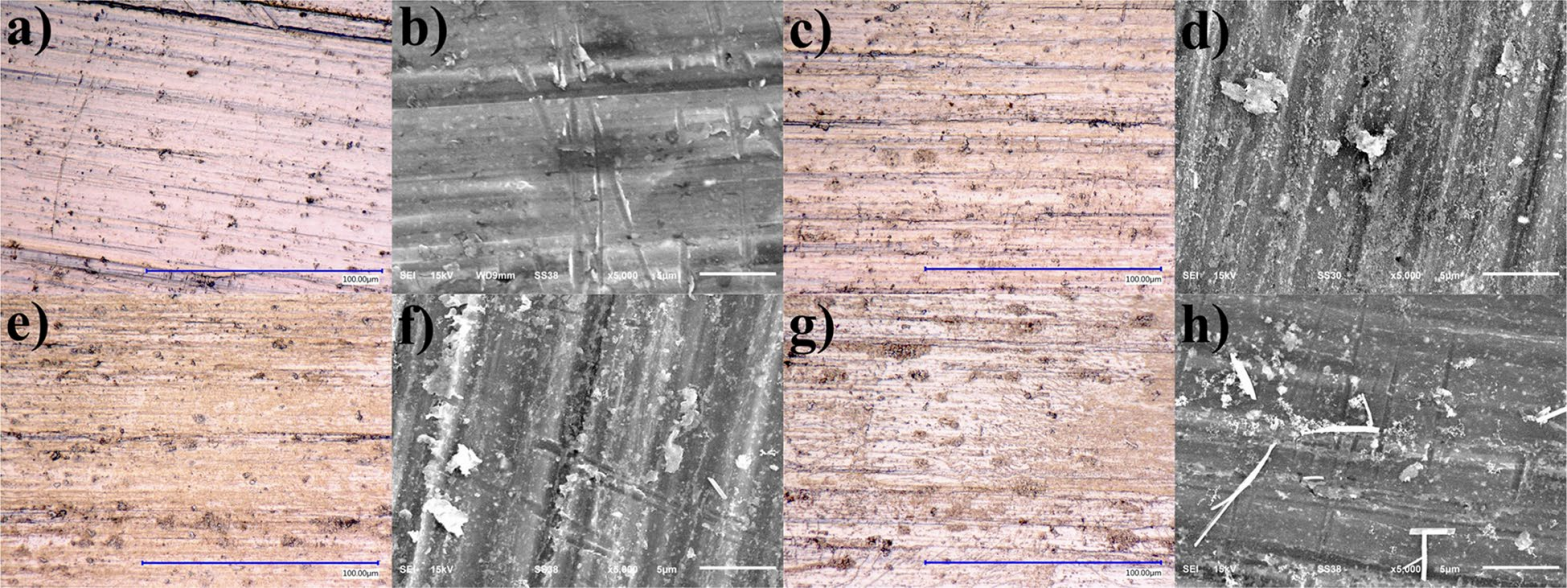
Optical micrographs at 2000× (scale bar 100 µm) and SEM images at 5000× (scale bar 5 µm) of samples immersed in Sm_2_(SO_4_)_3_ 8.4 mM at different times (**a,b**) 0 (without coating), (**c,d**) 20, (**e,f**) 40, and (**g,h**) 60 min, respectively.

Figure 2 shows optical and electron micrographs at 2000x of the samples immersed in Sm_2_(SO_4_)_3_ at 8.4 mM obtained by digital optical microscopy. A cleaned aluminum sample surface is shown in Fig. 2a,b. The growth of the samarium species particles as a time-dependent variable can be observed. On samples having 20 min of immersion time (Fig. 2c,d), there were slight changes in the color of the surfaces due to highly dispersed particles. The surface was covered by dispersed particles. The samples having 40 min of immersion time had an evident change in color and formation of a layer (Fig. 2e,f), which was attributed to the deposited samarium species. The most homogeneous layers occurred at 40 min. The samples having 60 min or more showed more irregular surfaces with large agglomerates. Also, the layers were observed with detachments (Fig. 2g,h). Based on the micrographs, the sample was selected at 40 min of immersion to conduct further tests.

**Figure 3:**
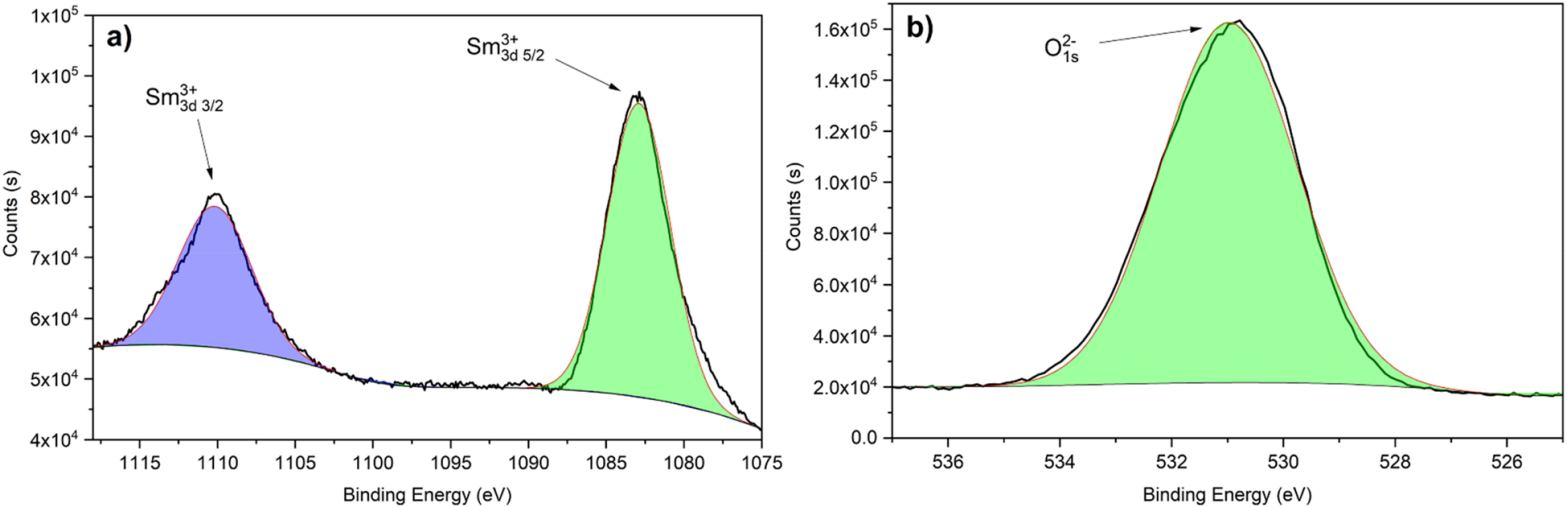
High-resolution XPS spectra of a samarium coating by immersion method corresponding to (**a**) Sm3d and (**b**) O1s.

The XPS analysis of Fig. 3 shows the composition of the species obtained by the immersion method, this spectrum can be deconvoluted into two signals according to their binding energy, attributed to oxide and other forms of metallic samarium^55,56^. In the Sm3d analysis, the signal found at 111.13 eV corresponds to Sm^3+^ 3d_3/2_ and the peak observed at 1082.84 eV was attributed to Sm^3+^ 3d5_/2_. In the case of the O1s analysis, a single peak was found at 530.81 eV, which was attributed to O^2-^. Based on these XPS results, Sm^3+^ and O^2−^ presence was attributed to Sm_2_O_3,_ which is stable at high pH values and can function as a protective barrier^57^. This confirms that the immersion method depositions favor the formation of Sm_2_O_3_. Nonetheless, this samarium oxide cannot significantly delay the corrosion of the aluminum since it is not in the form of a continuous layer.

### Samarium compound layers by APPJ

**Figure 4:**
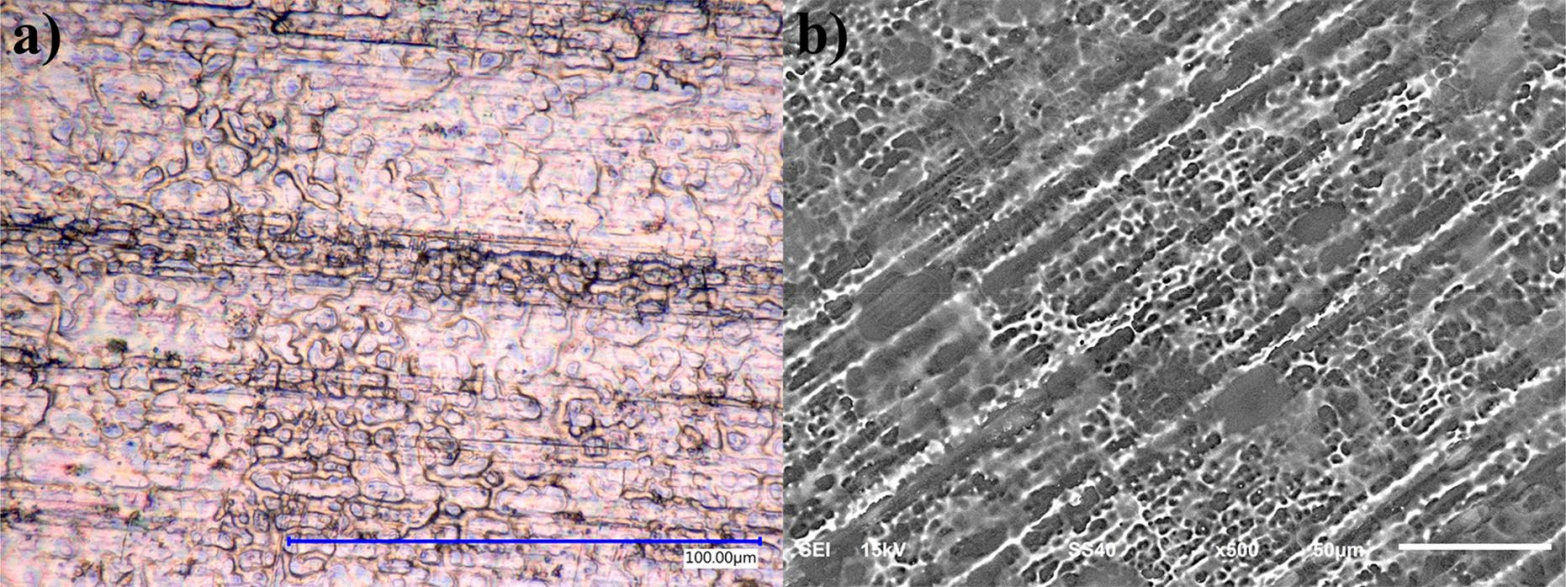
Aluminum surface with an Sm_2_(SO_4_)_3_ coating by APPJ observed using (**a**) optical (100 µm scale bar) and (**b**) electronic (10 µm scale bar) micrographs.

Figure 4 shows the coating obtained from exposing the substrate for 30 s to the plasma jet, using the ultrasonic fog of Sm_2_(SO_4_)_3_ as a precursor. A homogeneous and continuous layer is observed, generated because of the form that the plasma jet projects the species towards the substrate. This favors the coating deposition on the imperfections caused by the alloy polishing. The APPJ uses air as a propellant at a pressure of 5 bar generating plasma with both nitrogen and oxygen providing a rich environment of ions and temperatures up to 260 °C. This plasma propels the precursors in the ultrasonic fog toward the substrate, slightly increasing its temperature according to the mass in each droplet of the aerosol and the short time of contact in about 2 cm length. As a treatment method, the proposed result is a partial oxidation for some compounds that can be oxidized. The technique can drag and wrap some compounds or particles but using precursors, even atomic deposition can be attained by covering surfaces not directly exposed in front of the jet.

Figure 4a shows the optical micrograph at 2000× (scale bar 100 µm), which, compared to Fig. 2a, remarks the differences caused by the continuous layer on the aluminum substrate. The transparent characteristic of the coating makes it possible to identify the polishing direction lines. Also, an iridescent pattern is observed, attributed to a continuous layer causing interference with the visible white light, which, proceeding from the microscope, has a perpendicular incidence to the substrate. The observed surface morphology is characteristic of the formed transparent layer, independently of the original substrate finishing.

Figure 4b shows the SEM micrograph at 500× (scale bar 50 µm), which, compared to Fig. 2b, remarks the differences caused by the continuous layer on the aluminum substrate. It is possible to identify the polishing direction lines, but in this case, using electrons instead of photons to generate the micrograph. This image is challenging as we are looking at two surfaces at once. The outer surface is barely visible as clouds passing by a plowed cornfield, floating, and diffusely round shaped. The white streams follow the polishing direction on the substrate. It is proposed that the interface between the deposited layer and the substrate surface features dispersed this high intensity of electrons. The grey areas are proposed as a complete match and the white areas as a gap covering such features. This phenomenon did not appear in mirror finishing surfaces. Figure 4a,b are complementary.

**Figure 5:**
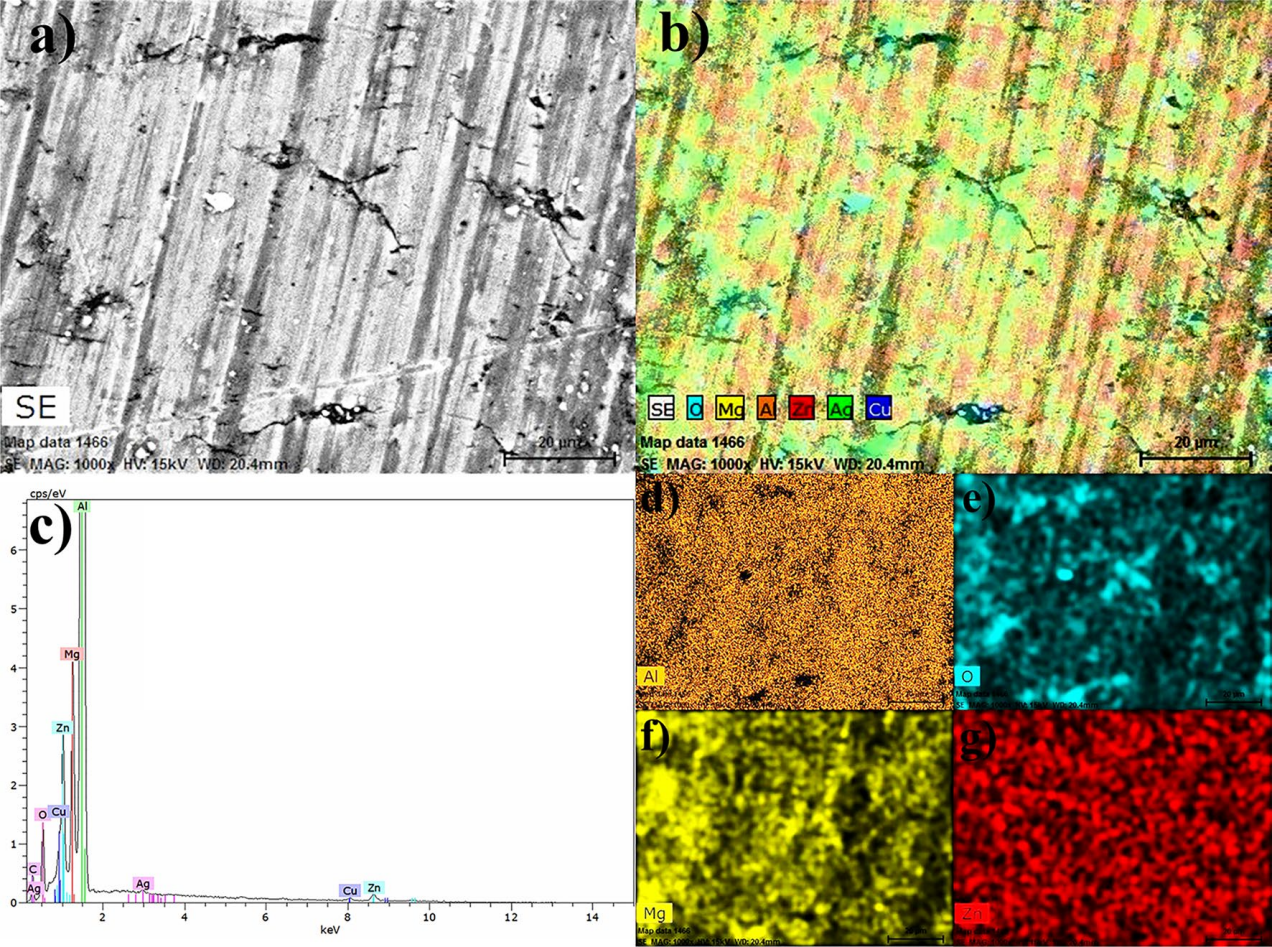
SME and EDS analyses of an aluminum 3003 cleaned sample.

**Figure 6:**
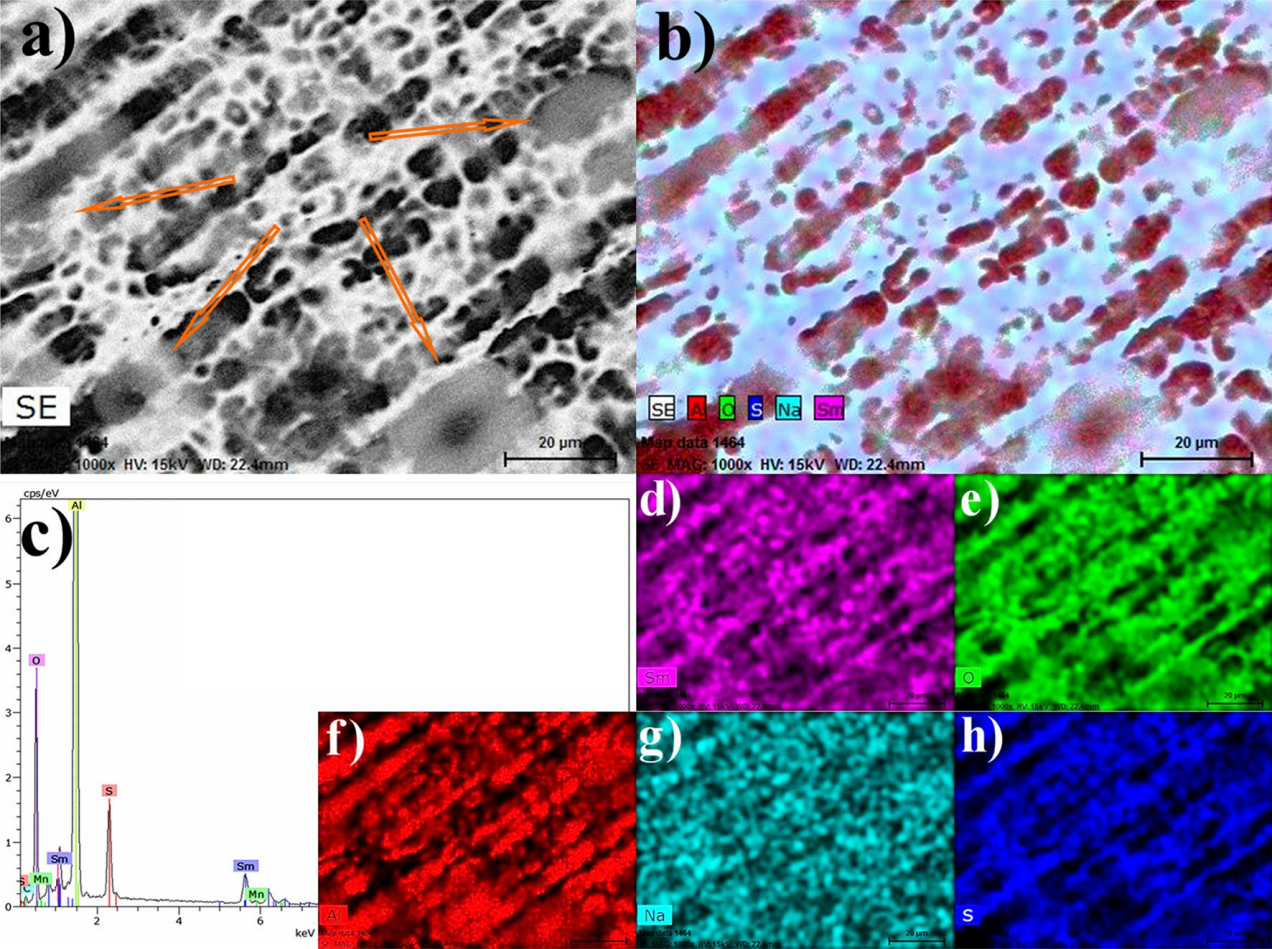
SME and EDS analyses of a samarium compound coating deposited by APPJ on aluminum 3003.

Figures 5, 6 and 9 are a series of EDS analyses conducted on three samples, bare aluminum 3003, the samarium compound layer by APPJ on aluminum, and silica coating on top applied using the APPJ technique with a fog of TEOS, respectively. In this series of micrographs, Figures a–c show the secondary electron images, the chemical mapping with diverse elements, and the spectra for such analyses, respectively. Table 1 resumes the quantitative analyses of EDS for the three cases. Finally, the three figures show the individual mapping with the prominent elements for each sample.

**Table 1:** EDS analyzes of aluminum (substrate), Sm_APPJ_ coating on AA3003 (single layer), and SiO_2APPJ_ coating on a Sm_APPJ_ coating on the AA3003 (double layer).

Element	Atomic number	Aluminum 3003			Sm_APPJ_/AA3003			SiO_2 APPJ_/Sm_APPJ_/AA3003		
		Norm. C [wt%]	Atom. C [at%]	Error [wt%]	Norm. C [wt%]	Atom. C [at%]	Error [wt%]	Norm. C [wt%]	Atom. C [at%]	Error [wt%]
C	6	8.5	17.02	1.77	3.71	7.93	0.98	23.57	35.32	2.61
N	7							3.31	4.26	0.58
O	8	6.22	9.35	1.06	22.63	36.26	3.19	36.97	41.59	3.71
Na	11				0.11	0.12	0.04	3.72	2.91	0.22
Mg	12	4.83	4.77	0.32				0.91	0.67	0.07
Al	13	75.05	66.88	3.94	50.71	48.19	2.6	0.4	0.27	0.04
Si	14							15.07	9.66	0.55
S	16				5.46	4.37	0.25	5.47	3.07	0.19
K	19							0.46	0.21	0.04
Ca	20							3.09	1.39	0.11
Mn	25				0.6	0.28	0.06			
Cu	29	1.21	0.46	0.1						
Zn	30	4.05	1.49	0.23						
Ag	47	0.13	0.03	0.04						
Sm	62				16.79	2.86	0.6			
Au	79							7.02	0.64	0.25

Figure 5 shows the SEM and EDS analyses for the bare aluminum alloy 3003 exhibiting the presence of Mg, Zn, Cu, and Ag (Table 1 linked to Fig. 5c).

Figure 6 shows the SEM and EDS analyses of a samarium compound coating deposited by APPJ. Figure 6a has orange arrows pointing toward the round shapes on top of the samarium compound coating. Figure 6b remarks that the previously mentioned in Fig. 4b as grey areas are red colored for aluminum. This is a composed image including a mixture with predominantly white areas for the secondary electrons. Sm is highly present with about 16.79 wt% (Table 1 linked to Fig. 6c). The EDS analyzes show the characteristic peaks of the presence of samarium on the surface^58,59^. Figure 6d shows the distribution of Sm on the surface and it is more similar to the oxygen image of Fig. 6e, which could be interpreted as a samarium compound having oxygen (oxide, hydroxide, or sulfate). Figure 6h, corresponding to sulfur, has similarities with these of Sm and O but also has some differences and a lower quantity with about 5.46 wt% (Table 1 linked to Fig. 6c). This could indicate that some sections were formed with compounds partially oxidized by interaction with the plasma and others without changing the solution content. These were dried and constituted a residue. Figure 6f corresponds to aluminum and is complementary to the others, occupying these areas vacant of the other elements. Sodium is more randomly distributed in Fig. 6g.

Figure 6, as a whole, can indicate the formation of some areas with samarium sulfate and others with samarium hydroxide or oxide. Samarium sulfate is contraindicated since it easily dissolves with water leaving empty sites. This can be prevented by reducing ultrasonic fog droplet sizes or samarium sulfate concentrations as process parameters.

**Figure 7:**
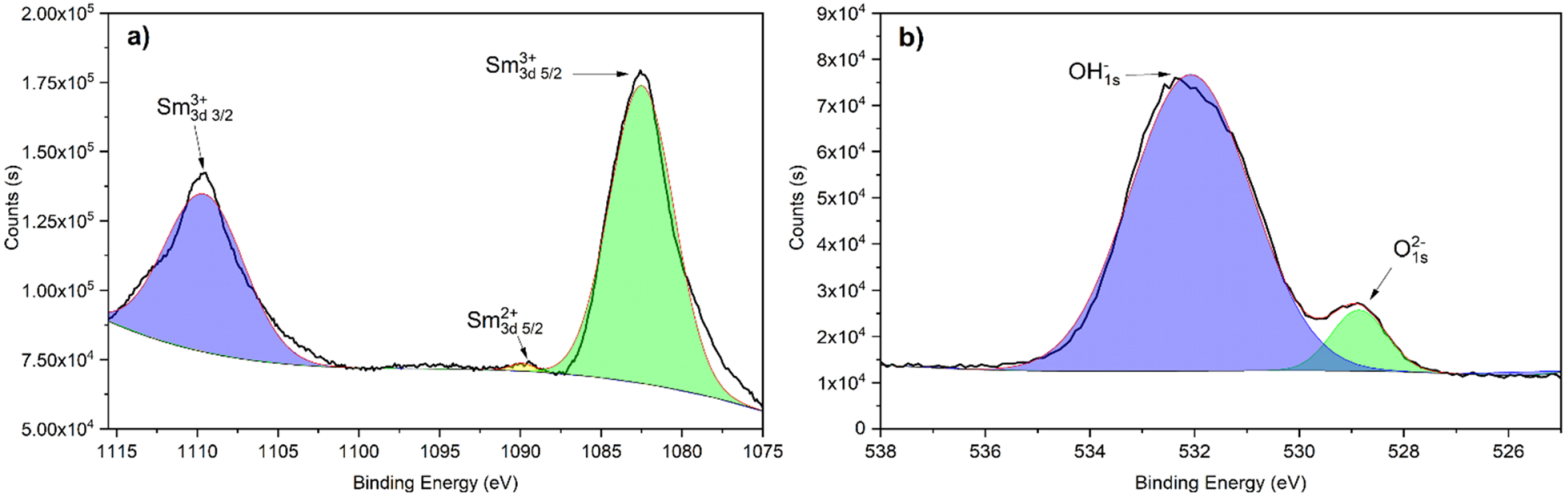
High-resolution XPS spectra of a samarium coating by APPJ corresponding to (**a**) Sm3d and (**b**) O1s.

Figure 7 shows the XPS analysis of the samarium coating obtained by the atmospheric pressure plasma jet method. In the case of the analysis of Sm3d (Fig. 7a), the spectrum shows the presence of Sm^3+^ and Sm^2+^ in the form of oxides and hydroxides, as reported by other authors for different samarium application methods^60–64^. Sm^3+^ peaks are reported at 1109.59 eV for Sm^3+^ 3d 3/2 and 1082.49 eV for Sm^3+^ 3d 5/2. And the Sm^2+^ is attributed to the peak found at 1089.92 eV. The presence of Sm^3+^ was associated with Sm_2_O_3_ and mainly Sm(OH)_3._ The Sm^2+^ signal corresponds to SmO. In the O1s analysis (Fig. 7b), the peak at 532.07 eV is attributed to OH^-^ and the peak found at 528.85 to O^2−^. This analysis shows that the amount of Sm(OH)^3^ increases by the APPJ method (compared with the immersion method) since the spectrum of O1s is mainly controlled by OH^−^. The increase in the presence of this hydroxide is a first indication of the possible higher efficiency of the plasma method to deposit samarium species as corrosion inhibitors since the hydroxide of samarium is considered a corrosion inhibitor^65^ and even a self-healing compound since its oxidation increases the volume and blocks the failures on the top physical barriers. In the case of the immersion method, the samarium hydroxide was not attained and in the APPJ method, the majority species is Sm(OH)^3,42^.

### Barrier layer of silicon dioxide by APPJ

**Figure 8:**
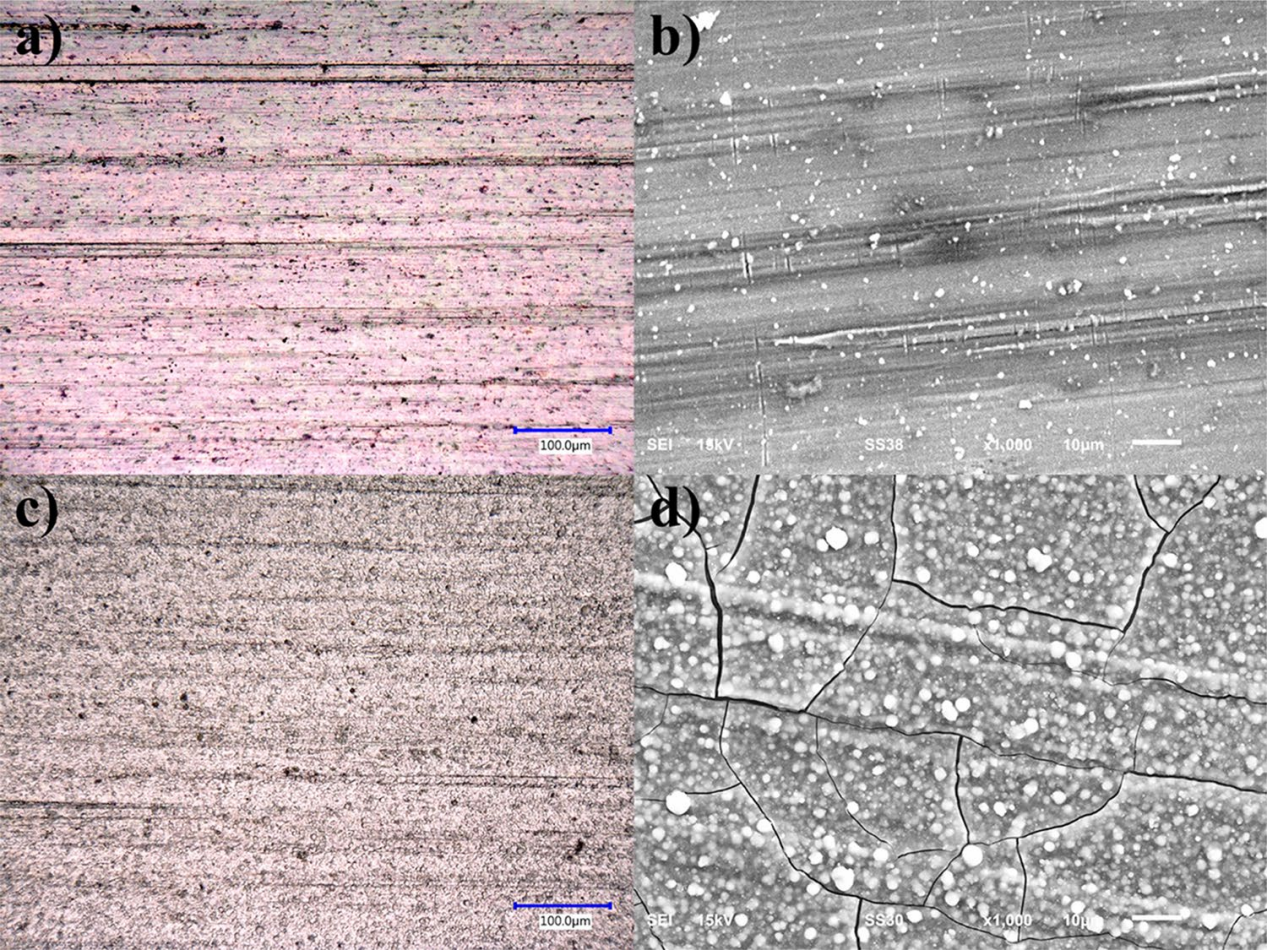
Optical (scale bar 100 µm) and SEM (scale bar 10 µm) micrographs of SiO_2_ coatings deposited by APPJ at different times (**a,b**) 60 s and (**c,d**) 70 s, respectively.

Figure 8 shows the SiO_2_ layer by optical microscopy developed as a function of the exposure time to the plasma. A thin layer was observed in the plates with a coating of 60 s (Fig. 8a). The colors are caused by an interference effect associated with the thickness and the refractive index of the coating^66^. Figure 8c shows a continuous layer using 60 s of SiO_2_ deposition using APPJ, exhibiting particles throughout the surface. A homogeneous thin film was observed in the plate with a coating of 60 s, which was continuously dispersed throughout the sample. From the layer of 70 s, a continuous layer covering the sample surface was observed. However, after 5 min of coating, the layer cracked and did not adhere correctly to the whole surface.

According to the results observed in the optical microscope, it was determined to take the samples exposed to APPJ for 60 s and 70 s to be observed by SEM.

Figure 8b,d show SEM micrographs of the SiO_2_ coating. Figure 8b corresponds to 60 s of deposition, which generated a continuous and homogeneous layer with a low number of agglomerates. Figure 8d, for a deposition time of 70 s, shows a layer with a higher number of agglomerates and fractures on the coating. Based on the results of the morphological characterization, it was determined that 60 s was the best time for the SiO_2_ layer since it fulfills the function of a barrier layer.

**Figure 9:**
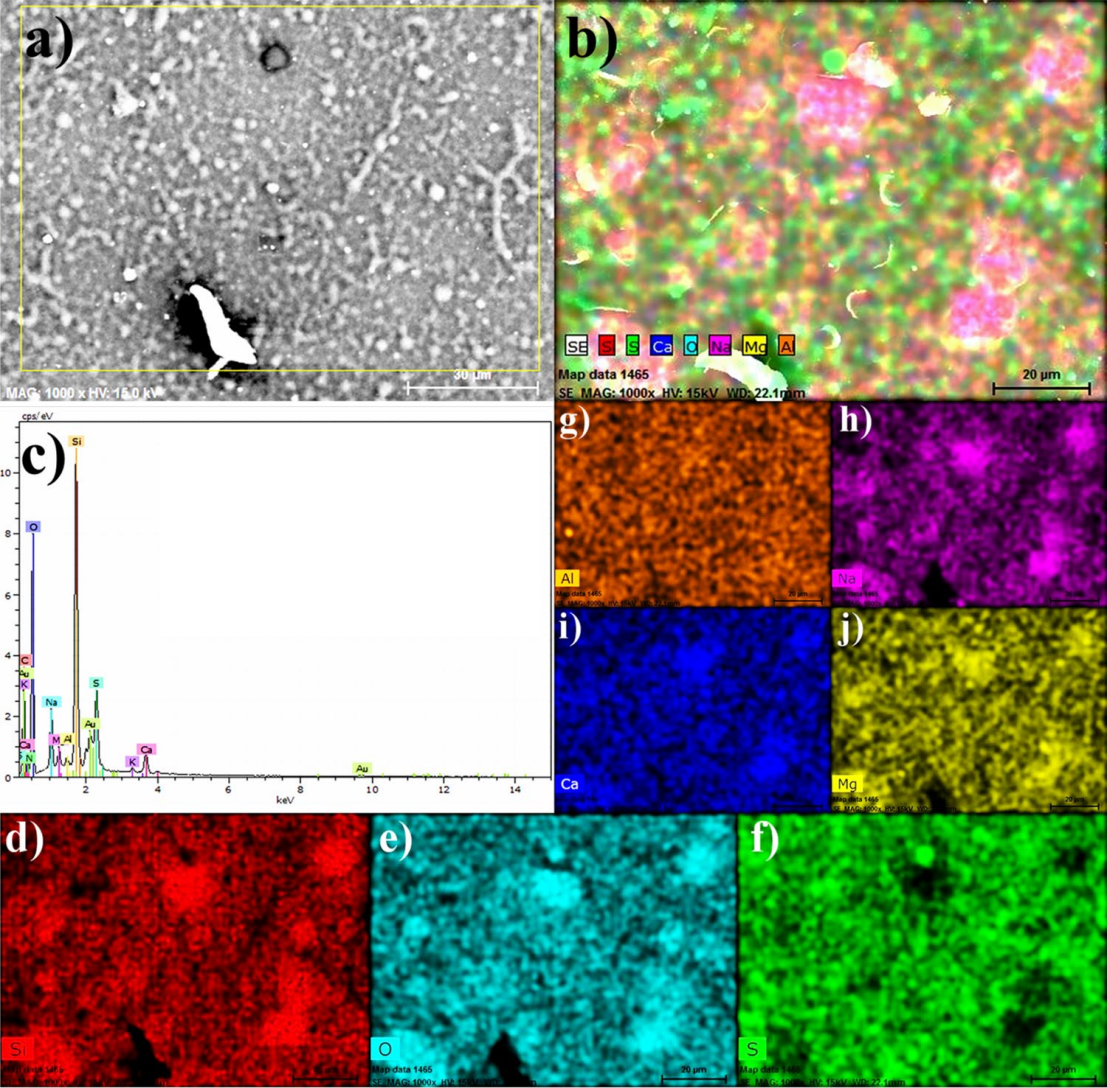
SEM and EDS analyses of silica deposited by APPJ and an ultrasonic fog of TEOS on a samarium compound coating by APPJ on aluminum 3003.

Figure 9 shows a silica coating on top applied using the APPJ technique with a fog of TEOS. Gold was used on top for better observing the surface with the electrons. Figure 9a shows the secondary electron image of the continuous silica coating with particles and interconnections between them. The homogeneity extends to the whole surface, providing a physical barrier for extensive areas. Also, it can be reproducible and for intensive industrial applications considering that the technique was installed on robotic arms. Figure 9b shows the chemical mapping with diverse elements. Again, similarly to Figs. 4b, 6a,b, this image is challenging as, probably, there is information that can be associated with any of the three interfaces. In this case, there is an interface between the substrate and the first coating by APPJ of samarium compounds, another for this coating with the second coating of silica by APPJ, and the outer one. Figure 9c, showing the EDS spectrum and Table 1, linked to Fig. 9c, with the quantitative analysis. Figure 9d–j, show the individual mapping with the prominent elements.

Figure 9, as a whole, can indicate a top layer of silica deposited by APPJ. This is based on the higher silicon content of about 15.07 wt% (Table 1 linked to Fig. 9c). Aluminum is randomly distributed as a background (Fig. 9g) with about 0.4 wt% (Fig. 9c). Similarly, sulfur is randomly distributed except for some spots. These previously observed cloud-type spots with diffuse round shapes appear as red (Si)–pink (Na)–blue (Ca)–magenta (O)–yellow (Mg) in Fig. 9c and individually in Fig. 9d,e,h,i,j. Samarium was not identified in these images, but sulfur in Fig. 9c was similar in quantity to Fig. 6c (5.47 *vs*. 5.46 wt%, respectively). Figure 9f is different in shape compared to Fig. 6h. All the other elements originated in the propelled solution interacting with the air plasma, basically nitrogen/oxygen. The EDS spectrum for the samarium coating obtained by APPJ shows the same peaks as similar systems obtained by other methods^67,68^. Therefore, the APPJ method could be an option for the deposition of samarium species.

**Figure 10:**
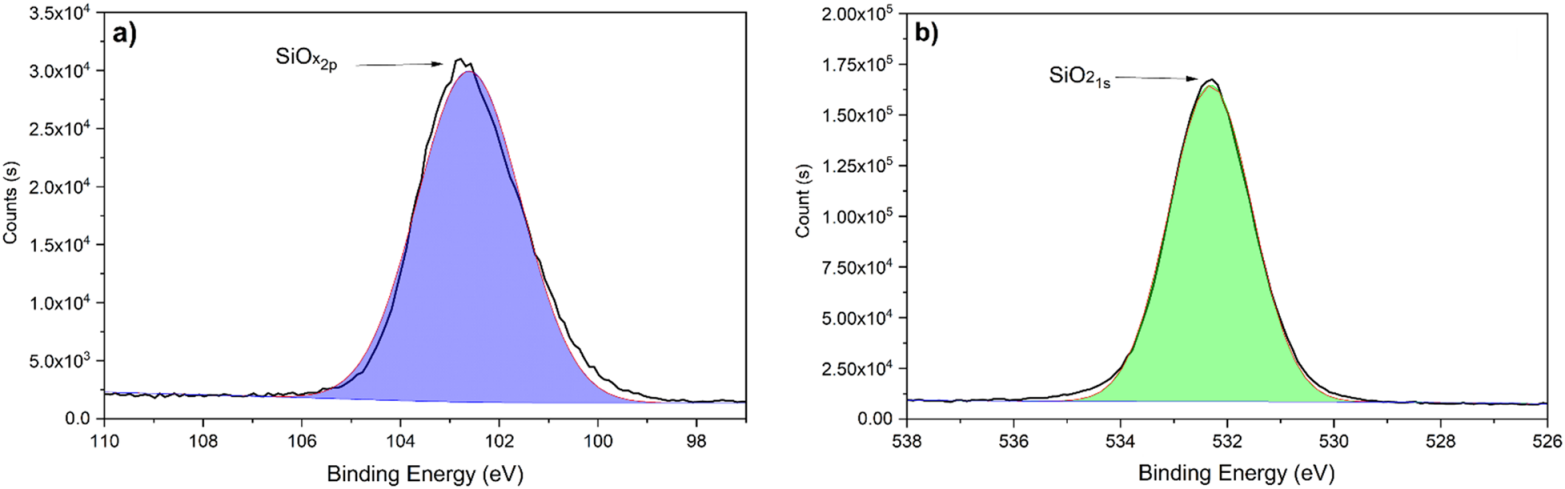
High-resolution XPS spectra of SiO_2_ coating by APPJ method (**a**) Si2p and (**b**) O1s.

The XPS analyses in Fig. 10 show the composition of the species obtained using a fog of TEOS in the APPJ. In the Si2p analysis, the signal found at 102.75 eV corresponds to SiO_x_ 2p. In the case of the O1s analysis, a single peak was found at 532.27 eV, which is attributed to SiO_2_ 1s. These results confirm that the obtained coating is formed by SiO_2_.

### Multilayer system

After the morphological and chemical characterization, it was determined that the best processing times for the electrochemical study of the multilayer system are 40 min for the immersion method and 30 s for the APPJ method, in the case of the layer of corrosion inhibitor. For the barrier layer of SiO_2_ by APPJ, the time was 60 s of exposure to the plasma jet.

**Figure 11:**
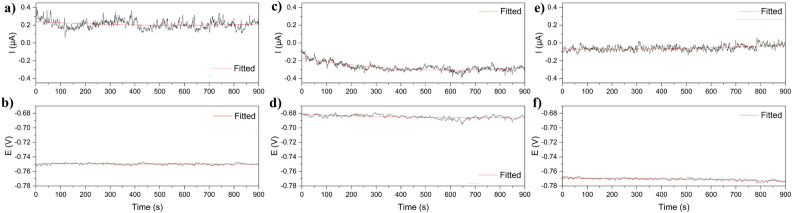
Electrochemical noise analysis for (**a,b**) aluminum without treatment, (**c,d**) Sm–Si_Im_, and (**e,f**) Sm–SiO_2 APPJ_ coatings.

Figure 11 shows the electrochemical noise measurements of aluminum 3003 without treatment (A_wt_), the multilayer system of samarium by immersion and SiO_2_ by APPJ (Sm–Si_Im_), and the multilayer system of samarium and SiO_2,_ both by APPJ (Sm–Si0_2 APPJ_). The potential and current variations are due to differences, between the two electrodes, in the initiation of localized corrosion, passive layer dissolution, and electrolyte permeation in the Sm–SiO_2_ coating.

In the Sm–Si_Im_ sample, the potential variations shift from − 0.75 in untreated AA3003 to − 0.68 V in the Sm–Si_im_. Both responses are attributed to lower electrochemical activity, caused by the passivity of the multilayer coating. In the case of the Sm–SiO_2 APPJ_ sample, the potential is more negative and the current approaches 0 µA. This can be attributed to the better conformation of the multilayer system, due to the continuous coating obtained by APPJ. This decreases the permeation of the electrolyte towards the coating.

Table 2 shows the results of Rn and PI obtained from the electrochemical noise measurements. The noise resistance is higher in the case of the coated samples, indicating less corrosion. The pitting index in all cases indicates localized corrosion, characteristic of aluminum.

**Table 2:** Electrochemical analyses showing the electrochemical noise, EIS, and Tafel results.

	Electrochemical noise		EIS			Tafel		
	Rn (Ω)	PI	Rs (Ω)	Rc (Ω)	Rtc (Ω	E_corr_ (mV)	I_corr_ (µA/cm^2^)	CR (mmpy)
A_wt_	20,875 ± 3485	0.45 ± 0.31	7.63 ± 0.84	–	37,990 ± 16,727	− 862.12 ± 94.12	0.131 ± 0.03	0.00070 ± 0.0002
Sm–SiO_2_ immersion	57,294 ± 30,104	0.31 ± 0.21	5.01 ± 2.05	10.27 ± 5.35	41,057 ± 5610	− 664.17 ± 12.29	0.5536 ± 0.59	0.0050 ± 0.0028
Sm–SiO_2_ APPJ	41,347 ± 15,667	0.51 ± 0.24	6.43 ± 1.96	9.88 ± 4.50	48,009 ± 25,434	− 830.65 ± 57.29	0.117 ± 0.08	0.00048 ± 0.0004

**Figure 12:**
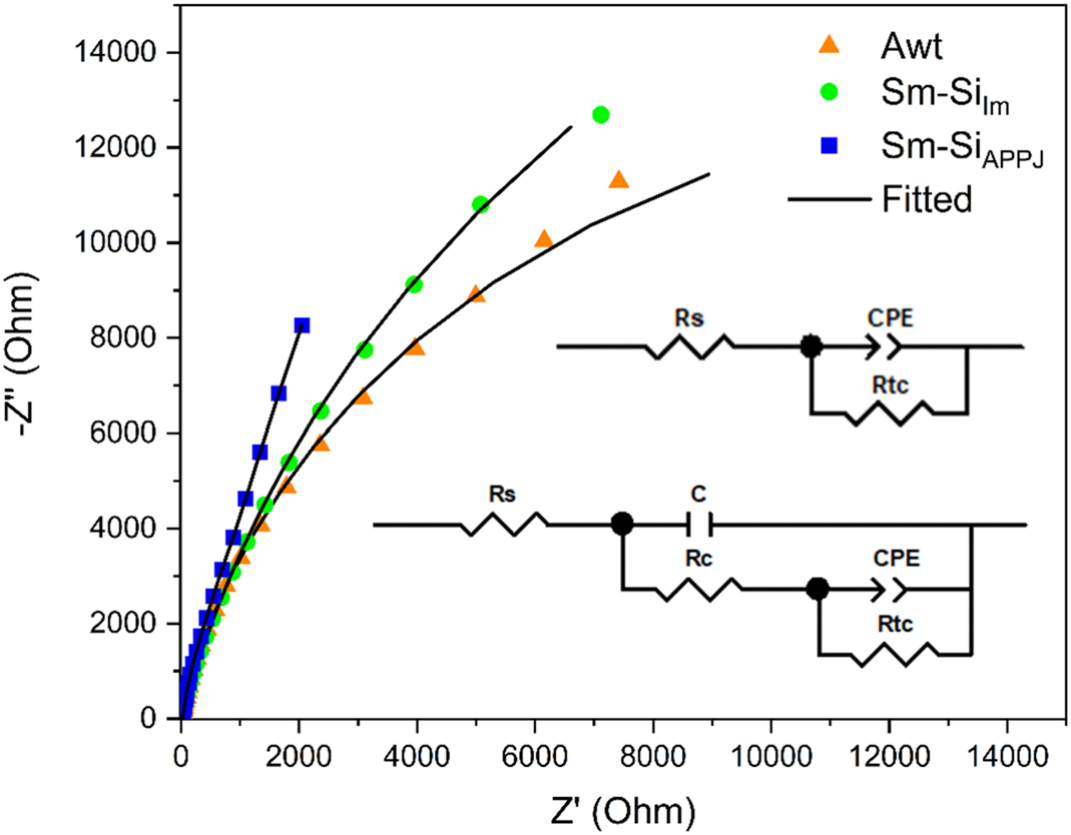
EIS analysis for aluminum without treatment and Sm–SiO_2_ coatings.

The electrochemical impedance spectroscopy analyses of Fig. 12 show two capacitive semicircles, the semicircles of the coated samples are larger than that of the untreated aluminum, which indicates a higher corrosion resistance. The best result was obtained in the Sm–SiO_2_ APPJ coating. The data obtained were adjusted to get an equivalent circuit (Fig. 12 insert). Equivalent circuits are a simplified representation of the electrochemical system and the chemical and electrochemical processes in the studied interface. The first one is a circuit system of three components which has been widely reported for untreated aluminum^27,69,70^. The second equivalent circuit, five components, is for coated samples and the two additional components are related to the electrical contribution of the coating^27,71,72^.

The components are electrolyte resistance (Rs), a constant phase element attributed to the electrochemical double layer (CPE), capacitance of the coating (C), resistance of the coating (Rc), and resistance to charge transfer. (Rtc), which is directly related to corrosion resistance. Table 2 shows the results obtained from the equivalent circuit, there is an increase in resistance to charge transfer in the coated samples. In the case of the Sm–Si_im_ coating, this result was attributed to a barrier effect. In the case of the Sm–SiO_2 APPJ_ system, it was attributed to a combined effect of the SiO_2_ protective barrier and the Sm corrosion inhibitor.

**Figure 13:**
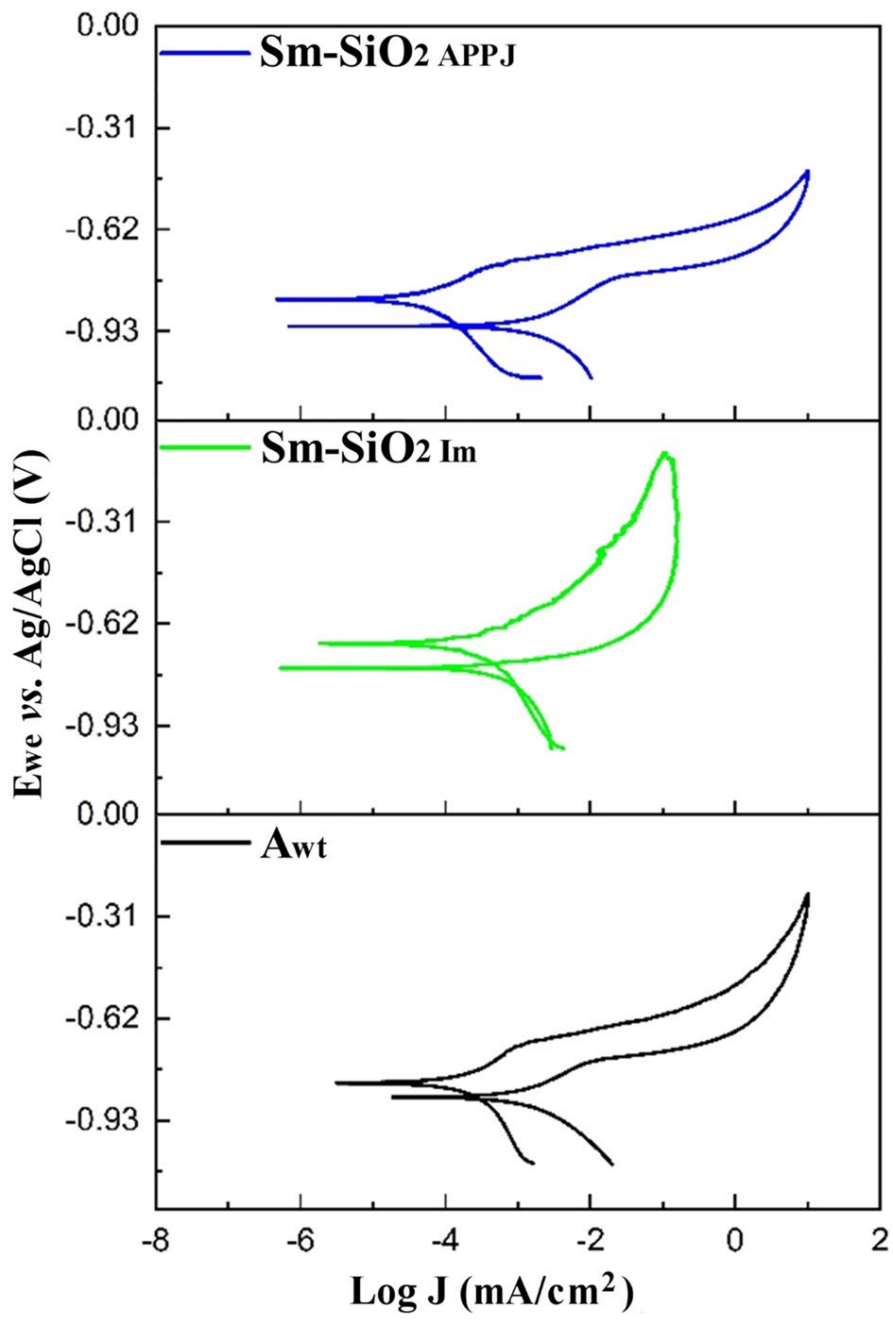
Cyclic potentiodynamic polarization for aluminum without treatment (A_wt_), Sm–SiO_2_ coatings by immersion (Sm–SiO_2 Im_), and APPJ (Sm–Si O_2 APPJ_).

Figure 13 shows the cyclic potentiodynamic polarization response for the untreated aluminum and coated samples. In all cases, the pitting potential is nearly equal to the corrosion potential and the formation of a hysteresis cycle is observed, which is attributed to localized corrosion^73^.

Table 2 shows the results of Tafel slopes. In untreated aluminum, the corrosion potential (E_corr_) is − 862.12 mV and the corrosion current (I_corr_) is 0.131 µA/cm^2^. In the case of Sm-Si_Im_ coating, the E_corr_ is up to − 664.17 mV and the I_corr_ is slightly higher. For the Sm–SiO_2 APPJ_ sample, the potential and corrosion current do not present significant changes. However, the corrosion rate is higher in the sample with the Sm–Si_Im_ coating, from 0.0007 mmpy in aluminum without treatment to 0.005 mmpy in the sample with a coating of samarium by immersion. The species of Sm^3+^ in the coating by immersion, being completely oxidized, does not work as a corrosion inhibitor, and contrarily seems to favor it. In the case of samples with Sm–SiO_2 APPJ_ coatings, there was a decrease of 31.42% in the corrosion rate. According to the results, the APPJ is a viable method to obtain a multilayer system to protect against corrosion.

The Sm–SiO_2 APPJ_ coating reduces the corrosion of AA3003 due to the combined effect of the continuous SiO_2_ layer and the Sm(OH)_3_ layer obtained by APPJ, which works as a corrosion inhibitor. The protection mechanism of the corrosion inhibitor is based on the oxidation of Sm(OH)_3_ to Sm_2_O_3_, the process is shown below^32^.

1. Aluminum oxidation:
  $$ {\text{3Al}} \to {\text{Al}}^{{{3} + }} + {\text{ 3e}} - , $$
  $$ {\text{O}}_{{2}} + {\text{ H}}_{{2}} {\text{O }} + {\text{ 4e}}^{ - } \to {\text{4OH}}^{ - } . $$
2. Because of the OH^−^ produced in the cathodic reaction, the pH increases at the electrode–electrolyte interface, and at that pH, the following reaction is favored:
  $$ {\text{Sm}}\left( {{\text{OH}}} \right)_{{3}} \to {\text{2Sm}}_{{2}} {\text{O}}_{{3}} + {\text{ 3H}}_{{2}} {\text{O}}. $$

Sm_2_O_3_ is formed preferentially in the cathode sites of aluminum 3003 and because it is stable under these conditions, it blocks these sites and thus reduces corrosion.

## Conclusions

The APPJ deposition of samarium compounds was attained and compared with a conventional immersion deposition method. A multilayer system was proposed using samarium compounds obtained for APPJ and immersion methods how corrosion inhibitors and silica coating by APPJ as a barrier layer to protect aluminum against corrosion.

The plasma jet method at atmospheric pressure allows us to obtain continuous and homogeneous coatings. The same air plasma jet was used as pretreatment for surfaces before deposition and to form the coatings by heating and partially oxidizing the compounds in aqueous samarium compound solutions or directly a silica precursor (TEOS). The solutions were put in contact with the plasma as an ultrasonic fog with varying deposition times. The first samarium compound coating was neither damaged nor detached by the second deposition and the obtained coatings were continuous and homogeneous.

XPS analyses show that the amount of Sm(OH)^3^ increases by the APPJ method compared with the immersion method since the spectrum of O1s is mainly controlled by OH. In the case of the immersion method, samarium compounds were completely oxidized, which makes that, at long immersion times, the samarium coating obtained is not viable to use as a corrosion inhibitor. For the APPJ method, the XPS analysis showed a suitable composition for its application as a corrosion inhibitor since the majority species is samarium hydroxide, this samarium hydroxide can be oxidized under conditions corrosive to aluminum and form insoluble oxides that inhibit corrosion by blocking cathode sites.

EDS analyses indicated the formation of some areas with samarium sulfate and others with samarium hydroxide or oxide. Samarium sulfate is contraindicated since it easily dissolves with water leaving empty sites. This can be prevented by reducing ultrasonic fog droplet sizes or samarium sulfate concentrations as process parameters.

It was determined that the best processing times for the electrochemical study of the multilayer system are 40 min for the immersion method and 30 s for the APPJ method, in the case of the layer of corrosion inhibitor. For the barrier layer of SiO_2_ by APPJ, the time was 60 s of exposure to the plasma jet.

The Sm–Si_Im_ system favored corrosion due to the excess of samarium oxide. On the other hand, the multilayer coatings obtained by the APPJ proved to have an anticorrosive effect reducing the corrosion of AA3003 by 31.42%. This is because of the combination of the protective barrier influence of the SiO_2_ layer and the effect of the samarium corrosion inhibitor.

## Data Availability

The datasets used and/or analysed during the current study are available from the corresponding author on reasonable request.
